# Book and Pamphlet Notices

**Published:** 1860-09

**Authors:** 


### Editorial.

### BOOK AND PAMPHLET NOTICES.

WALSHE ON DISEASES OF THE LUNGS; INCLUDING THE PRINCIPLES OF PHYSICAL DIAGNOSIS: A new American, from the third revised and much enlarged English edition. Blanchard & Lea, Philadelphia, 1860.

We are gratified in being able to announce another edition of this now standard work. It is scarcely necessary to say that the topics discussed are traced by a masterly hand—the previous editions have sufficiently acquainted intelligent readers with the abilities of Dr. Walshe, and the zeal, large experience and facile pen which he brings to the work before him. The present edition is not merely a reprint, but as the author remarks in his preface, has been carefully revised and much enlarged, and may be said, in the main, to be re-written.

Part I treats especially of the Clinical Topography of the Chest, and the Methods of Physical Diagnosis.

Dr. Walshe's analysis of the subject of Physical Diagnosis, whilst it may not be said to afford anything of a strikingly original character, nevertheless epitomizes and excludes, in a manner it is really refreshing to notice in these days when medical book-writers are so prone to aspire to bulky volumes with necessarily diffuse, verbose, and cloudy descriptions.

We would call attention to the author's remarks upon the subject of mediate and immediate auscultation. He evidently does not place large dependence upon the stethoscope.

" Persons, indeed, are to be found who seem to think that the stethescope possesses some mystic faculty of communicating diagnosis, and saving the auditor all trouble of thought. Unfortunately the instrument is no more than—

"Das todte Sprachrohr, das den Schall
Empfangt und wiedergeibt, und selbst nicht horet."

"The motto of the stethoscope might aptly run;

. . . . fungar vice cotis, acutum
Reddere quae ferrum valet, exsors ipsa secandi."

It is said that sometimes immediate auscultation is "disagreeable or indelicate;" we never could see why the thinnest fabric of cloth, (provided it be clean,) would not relieve this difficulty as well as a billet of wood or a miniature hose pipe. Custom, however, must govern, but it should ever be recollected, as the author observes, that "it is as necessary to try on a new stethescope, as to try on a new hat."

Dr. Walshe disagrees with Skoda on the possibility of diagnosing superficial and deep-seated sounds within the chest; and also thinks Dr. Flint's suggestion that bronchial and cavernous respiration may be distinguished by the relative pitch of inspiration and expiration, to be liable to so many exceptions as to be "unworthy of trust."

Among the most valuable sections in this part of the week is that with reference to "Pressure Signs," (§ 487 *et. seq.*) Under this head he gives "a succinct account of certain physical effects, produced on the wall of the thorax, on its contents, or on parts adjoining; and which are clearly due to pressure exercised from within the chest. Some of the most positive and easily ascertained evidences of intra-thoracic disease, are furnished by these pressure signs."

Our space will only permit us to call attention to the pages, although we should be happy to reproduce them entire.

On the whole, we are compelled to say, that we have not, in a long time, read a more sensible, perspicuous, concise, and practical one hundred and fifty pages on "Physical Diagnosis," than those of Dr. Walshe's Part I. We rank it with Dr. Barclay's perspicuous and elegant elucidation of the same topic, in his invaluable treatise on "Medical Diagnosis.

Part II is devoted to a clear and thorough exposition of Diseases of the Lungs and Appendages. Among the diseases mentioned, are pleurodynia, intercostal neuralgia, bronchitis and its varieties, organic changes of the bronchi, tuberculization, and other structural changes of the bronchial glands; the various disease of the pleura, with results; pulmonary neuroses, primary morbid changes of the lungs, condensation, hypertrophy, etc.; pneumonia, and its varieties; œdema, gangrene, cirrhosis, hemorrhage, apoplexy, hemoptysis, adventitious deposits; chronic consumption, progressive and retrogressive; acute consumption, primary and secondary; hæmatoma, carcinoma, cancer, entozoa, asthma, influenza, pertussis, mediastinal tumor, and abscess.

From this catalogue it will be seen at a glance that the author has gone very thoroughly into his subject, and from a careful examination in detail, we are prepared to say that his method is, in every respect, a model one. His descriptions are forcible and aphoristic, and diagnosis clearly and strongly defined. In the treatment of pleurisy, bronchitis, and pneumonia, in the acute forms, we notice that Dr. Walshe still insists upon the propriety of moderate venesection, rarely carried, however, beyond twelve or fourteen ounces, even in athletic adults. The whole scheme of treatment is based on anti-phlogistic measures, differing but in a few details from standard methods. Recognizing the occasional utility of blood-letting, in these acute inflammatory affections, nevertheless we are convinced that, in the large proportion of cases of the kind, to be observed here in the Northwest, practitioners find it wholly unnecessary, although, technically, it may be "well borne."

It is noteworthy, considering the great importance attached to the use of veratrum viride in these 'esions by not a few practitioners in this country, that our author, who is evidently familiar with American books, does not even mention it among curative agencies. But that time-honored sedative, tartarized antimony, he observes, "stands next in importance to venesection in the treatment of pneumonia. Were I, indeed, henceforth, in the management of this disease, forced to surrender either, on the one hand, venesection; or, on the other, cupping and tartarized antimony; I should not hesitate to relinquish the former."

Another observation with reference to pneumonia is worthy of notice: (§1160.) "The announced success of treatment by copious libations of brandy appears simply to furnish a fresh illustration (as conversely Bouillaud's alleged triumphs by his *Saignees coup sur coup* in genuine typhoid [peyerian] fever) of the *vis medicatrix naturae.*"

About seventy pages of the work are occupied by the subject—consumption, and these may be considered the most valuable part of the book.

The author, in common with the best observers of the present day, is not disposed to abandon the phthisical patient as an *incurable.* For striking illustrations of the truth of his position we would call attention to § 1518, p. 387, which space will not permit us to quote entire. *Specifics* are considered as unreliable—"but cod-liver oil cannot be so lightly dismissed as it more rapidly and effectually induces improvement in the general and local symptoms than any other known agent, although its power of *curing* the disease is undetermined." The brown oil is preferred, and the other kinds of oil, almond, cocoanut, neat's foot, &c., are characterized as failures. Glycerine, however is admitted to be an important and often eligible substitute. The phosphate of lime, and Dr. Churchill's hypophosphites are very properly passed over with scant and uncomplimentary notice. Hygienic measures are mainly relied upon, and as given, are such as, in the main, command approval. Whilst cautious in his expressions, nevertheless he inclines to approve the moderate employment of alcoholic beverages in the progress of the disease in some of its forms. The exceptional cases which he suggests, are so few as scarcely to make against the general rule.

We cannot forbear noticing, in this connection, the author's reference to the relation of phthisis to the marriage contract. Will a phthisical condition undiscovered until after a matrimonial contract is entered upon, on its subsequent discovery, afford a legal bar against prosecution for its non-fulfillment by the patient? What an opportunity here presents itself for professional ventilation of stethescopic skill in future breach of promise cases. *Horresco referens!*

We should like much to follow Dr. Walshe in his further remarks upon phthisis, upon asthma, influenza, and pertussis; but, unfortunately, that which we had intended only as a brief notice has already expanded into undue proportions, and we abruptly close by again commending this valuable work to the shelves and to the perusal of the reading members of the profession. A.

---

Electro Physiology and Electro Therapeutics; showing the best methods for the medical uses of Electricity, By Alfred C. Garrett, M. D., Fellow of the Mass. Medical Society, Boston. Ticknor & Fields, 1860. Received through S. C. Griggs & Co., 39 and 41 Lake Street.

According to forerunning announcement this volume comes to us in unexpected proportion, in goodly apparel and attractive type. It adds not a little to the *readability* of the work, that its mechanical execution is so good that it has attractions for the eye as well as for the mind. Our author does not profess to walk in new or untrodden paths, but "aims to confine himself to gleaning from the highest practical authorities, and the comparing of these with his own clinical experiences, then classifying and arranging the subject matter so as to present the whole range of electro therapeutic practice on a more systematic and scientfic basis." He says: "The very abridged manner and immethodical style of the few works that have appeared in this country, or even in the English language, on the medical employment of electricity, have never yet enabled the medical profession generally, partiularly in this country, to seize upon these telling facts understandingly, so as to bring them to bear upon clinical practice." Such a gleaning from the storehouse of the past, such a reformation in style has Dr. Garratt attempted, so as to bring existing knowledge within available compass, and render it inviting to members of the American medical profession.

We confess ourselves somewhat stumbled on opening at the preface and finding that "Life is electro-chemistry *vitalized*," but suppose ourselves wanting in metaphysical inclination. What essential position the "electro" may occupy in the phenomena of *life*, we are not disposed at present to inquire. We propose only, in narrow limits, to notice the leading features of the work.

The first chapter, treating of Natural Electricity, gives a very concise view of electric laws and phenomena, as explained and understood at the present time. It serves to revive one's knowledge of that branch of natural science, and freshen our school-day impressions. We are not, however, prepared to accept the implied teaching that "Electric changes are the causes of epidemic diseases." While the author quotes from M. Andraud and Dr. Kidd, and also gives Sir James Murray's fifty reasons therefor, he is somewhat careful about committing himself to a theory that finds but few advocates among the observers and writers in our profession at the present day. But we must leave these points for those who may read the book.

"The early history of the medical uses of electricity," constituting chapter second, is replete with interest; tracing galvanism, in its history, from Galvani to the present time. One is led to not a little sympathy for that martyr to galvanic science—poor frog—who has suffered martyrdom for the development of galvanic science for the past three-fourths of a century. The first three chapters form the natural history of the author's therapeutical agent.

Chapter fourth seems more properly to form the basis of the work, and however readily we may admit all the author claims as the therapeutical effect of electricity, we frankly confess we fail to perceive the force of much of the reasoning that aspires to important conclusions, and makes this a long chapter. The bias of the author may be perceived in such phraseology as "nerve battery," "brain battery," as applied to the brain; "sub battery." as applied to the ganglia; while muscle is exalted into "a kind of *minute compound voltaic battery* of membranous cells." Of the brain he says: "It is now manifest that almost every structure and arrangement here found may be nearly imitated by voltaic arrangements, combinations, and manifestations."

Of the blood vessels and muscular system, he avers, "Each of these muscle fibres is completely enveloped by the minute blood-vessels which run parallel with them. This admirable arrangement of the capillaries yields the abundant supply of arterial blood so necsssary for the manifestation of muscular motion; as contractions evidently ensue from changes taking place in the material in, of, and about, the ultimate fibre by a nervo-voltaic process; and also, perhaps, by artificial galvanism." From such premises he affirms, "The actual bearing of these investigations shows that in whatever process or function of the human body the blood is the most essential, these, nothing less than true voltaic or animal electric actions, and *life force* must have an influence. Furthermore, since it is self-evident that the circulation of the blood has thus to do, more or less, with every operation of the living body, I will only add that these experiments and considerations demonstrate the vast importance of employing artificial electricity, if possible, as a co-worker with nature's similar actions in our organisms, as a rational, natural, and extensive remedial agent." Or, in other words, since the structure and arrangement of the brain may be imitated by voltaic arrangements, and since muscle is "a kind of minute compound voltaic battery," and as muscular contractions follow as a result of a nervo-voltaic process, therefore follows, as above, a demonstration of "the vast importance of employing artificial electricity, &c." We simply dissent.

There is much reasoning of which we fail to see the force, since we do not admit there is anything physiologically essentially common between electricity or magnetism and nervous influence.

As soon would we reason a similarity between calomel and ordinary food because they will each pass the alimentary canal, excite peristaltic motion by muscular contraction, while the former is claimed by its admirers to exert stimulant, sedative, alterative and other influences as numerous as the most ardent electrist would presume to claim for his agent. In short, we firmly believe that any argument, based on any such supposed analogy, is without a sure foundation, and any therapeutical

*principles* thereby derived are useless, however successful the *practice* may be.

The therapeutical application of electricity, constitutes the last and larger part of the work, and herein is the value of of the work; herein is what makes the book such us may be, with profit, in the hands of every practitioner of medicine. We doubt not here is a field, valuable and unexplored by a very large majority of medical men. There are practical facts and teachings here that every one should know, even if he does not make the subject a speciality.

There is, perhaps, a lack of that clearness and conciseness in stating cases, that we are accustomed to find in our leading medical literature; but the truth is there and deserves to be known. The reader will also notice a rather free generalization, in some parts, especially regarding treatment, that renders the book of less practical value. The author may thank, or forgive us as he may feel disposed, but we must quote his treatment of epilepsy. After quoting page by page from Marshall Hall, and theorizing for almost twenty successive pages on "spastic diseases," the sum of treatment is as follows:

"As regards the treatment of epilepsy, and of kindred affections, we are to direct the most powerful *modification of nervous nutrition*, whether electric currents shocks by successions of hot and cold water; by nervo-polarizing, or depolarizing medicines, as iron, iodine, and sulphur; or silver, arsenic, strychnine, and quinine; or by opium, conium, stramonicum, nicotine, and belladonna, in some way, and by all means, so as bear, directly or indirectly, on the identical spot or part of exalted excitability of both peripheric nerves and nervous centres, to strengthen, and hence to calm and fortify them."

Now we are friends of "general principles"—we were reared in medicine when such were taught; but we must remonstrate here. Reader, re-read the foregoing, and say if it is not comprehensive enough for all; but if you attempt a case of epilepsy you will be thrown somewhat on your own resources, or, in despair, send your patient to Dr. Garratt, who, comprehending the above, will doubtless cure "in some way, or by all means."

The author's treatment of ague is more explicit, and we venture to subjoin it, hoping to see any case of successful treatment reported:

FIRST, THE PLACE.—The patient, we presume, is in bed, in a room where the air is dry and warm, (not sitting up before the blaze of a fire, nor where a draft of wind is allowed to blow upon any portion of his body or limbs.) SECOND, THE TIME.—Just a little before the hour and minute for the invasion of the cold stage, shake, or rigors, is the time to work. Prepare the patient comfortably—wet the sponge electrodes in *very warm water*, or salt water, and see that the battery or machine works well; then commence *before* the *horripilatio* of yawning, stretching, sighing, "cold chills down the back," goose-flesh, and collapse of the features, *get the start*, (as in Faradaic tooth-extracting,) and maintain it, first by a current from the nape of the neck to the regions of the liver, stomach and bowels; next up and down the back; and then using *team electrodes* to Faradize the surface. When the chill is broken, repeat a daily *seance*, but at different hours, to fortify the invigoration."

We have extended our "notice" farther than at first designed. We commend the work to careful readers, and to the judgment of the profession it seeks to enlighten.

L. S. E.

---

NEW MEDICAL JOURNAL.—We have received number one of volume one, of the *Columbus Review of Medicine and Surgery*, published at Columbus, Ohio, W. L. McMillen, M. D., being its editor and proprietor. It is a bi-monthly of 96 pages, and is furnished to subscribers at two dollars per annum, payable in advance. In its mechanical execution the work exhibits a high degree of excellence, and the brain-work shows both capacity and a willingness to labor. The first number contains elaborate reviews of Todd on Acute Diseases, Draper's Physiology, and Stille's Therapeutics, written with much ability, and a clear appreciation of the merits of these works, and the other articles are of general and scientific interest to the profession. We trust that eminent success may attend this enterprise so auspiciously inaugerated.

